# Cancer prevention and control: Kaposi’s sarcoma

**DOI:** 10.3332/ecancer.2019.951

**Published:** 2019-07-25

**Authors:** Jackson Orem

**Affiliations:** Uganda Cancer Institute, Upper Mulago Hill Road, PO Box 3935, Kampala, Uganda

**Keywords:** HHV8, AIDS-KS, Kaposi’s sarcoma, HIV, KS, KSHV

## Abstract

Kaposi’s sarcoma (KS) is a vascular tumour of endothelial origin that is associated with human herpes virus-8 infection. In sub-Saharan Africa, AIDS-KS remains the most common HIV-associated malignancy, and hence it poses a huge burden to the already constrained health-care systems. KS has four clinical variants, namely, classic, endemic, iatrogenic and epidemic KS. The histopathology in these different KS forms is essentially identical; however, they have different clinical patterns. Expanding knowledge of KS biology increases hope for prevention, disease control, and hence better quality of life among patients. Primary prevention strategy for KS-associated herpes virus and management of disease complication, such as lymphoedema should be the focus of disease-prevention and -control research.

## Introduction

Kaposi’s sarcoma (KS) is a multifocal angioproliferative disorder of vascular endothelium [1]. There are four clinical variants of KS: classic, endemic, iatrogenic and epidemic KS [2]). The classic form of KS was originally described by Moritz Kaposi. This is an aggressive tumour of older men of Mediterranean or Eastern European origin mainly affecting the skin. The endemic form of KS was described as early as 1963 before the advent of HIV [3]. Also called African KS, this form is aggressive and affects both adults and children. The substantial occurrence of KS in African children reflects the high level of KS-associated herpes virus (KSHV) infection in the population [4, 5]. With the emergence of HIV, the AIDS-associated KS is now the most common variant of KS in sub-Saharan Africa [6]. Iatrogenic or transplant associated KS is not common in Africa due to the low levels of transplants currently done on the continent, but this could become a problem in the future.

## Aetiology

For decades, the aetiology and pathogenesis of KS was unknown. In 1994, Chang et al. [7], reported the discovery of the KSHV, also known as human herpes virus-8 (HHV-8), and demonstrated an etiological link between the virus and KS. KSHV induces angiogenic and inflammatory cytokines, as well as gene products implicated in angiogenesis [8]; HIV infection further potentiates the development of KS through the transactivation (Tat) protein, which acts as a growth factor for KS [9]. The Tat protein induces endothelial cell proliferation and facilitates the invasion of extracellular matrix [10, 11].

## Histopathology

Although KS lesions may be clinically typical, biopsy of the lesion is needed for diagnosis and before patients are subjected to cytotoxic treatment. The histologic features characteristically comprise of large endothelial cells spindle shaped tumour cells. Presence of erythrocyte extravasation, inflammatory mononuclear cell infiltration, lymphocytes macrophages and plasma cells tend to be diagnostic [12]. Although the histopathology of the different types of the Kaposi tumour is essentially identical in all of these groups, the clinical manifestations and course of the disease differ dramatically [13, 14].

## Clinical presentation

KS most frequently presents as cutaneous lesions, which may occur in any site on the body [13]. The lesions are usually linear and symmetrical following creases: They are hyperpigmented, ranging from brown to black in Africans and other shades, including pink to purple depending on race [15]. They may be flat, plaque-like or nodular and vary in size and dimensions [16]. However, absence of cutaneous lesions does not exclude visceral KS. The next site after cutaneous are oral lesions usually appearing as purple or brownish plaques on the soft palate or gingiva which are in most cases asymptomatic. They may, however, ulcerate, get infected or bleed and may be painful. Oral lesions often point to the presence of lesions in other parts of the gastrointestinal tract or viscera [17]. GI and lung lesions are frequent sites of bleeding causing anaemia or frank haemorrhage. Pulmonary KS is a common site of extracutaneous involvement, and can be life threatening [18, 19].

## Diagnosis

The most common differential diagnosis of cutaneous KS lesions in Uganda is bacillary angiomatosis; tissue evaluation is important in differentiating the two. However, microscopic features are quite similar showing abundance of proliferating mononuclear inflammatory and spindle cells, ill-defined vascular channels, haemorrhage and oedema. The best differentiator is, therefore, additional tests for KSHV DNA which is negative in bacillary angiomatosis [20]. Since the emergence of AIDS, diagnosis of KS mandates evaluation for the presence of co-existing HIV. The HIV sero-positive patient requires evaluation for opportunistic infections, and immune functions (CD4 and viral load). Initial workup for staging AIDS-associated KS involves a complete physical examination, imaging (chest X-ray, an abdominal ultrasound scan), chest X-ray in particular is useful in excluding other lung pathology and lesions, such as tuberculosis, in low-resource settings. Other tests, such as faecal blood test, complete blood count, liver function tests and renal function tests, are important basic investigations. Endoscopy or bronchoscopy may be needed in suspected pulmonary or gastrointestinal disease.

## Prevention and control of disease

Currently, there is no vaccine against KSHV and no primary prevention. Given the lack of knowledge of the route of acquisition, no proper prevention message can be provided to the public. Riding on the back of the HIV prevention success, the measures in place for HIV can be used in the prevention of a KS epidemic. From an epidemiological perspective, KSHV presents a case for preventable vaccine for stopping transmission.

From the clinical perspective, since the majority of tumour cells are latently infected with KSHV, lytic replication plays a major role in disease progression and virus dissemination [21]. Therefore, transformation to lytic cycle could be a preventive strategy by increasing targets through augmented cell replication. Clinical trials to date have been negative for most inhibitors of DNA polymerase, such as ganciclovir, cidovir and foscarnet; hence, such agents should not be used outside of a study setting [22].

At a population level, a key preventive strategy would be breaking the transmission of KSHV through exchange of saliva.

## Control of active disease

Currently, KS has no cure. The goals of treatment are to control disease symptoms and prolong life, and therapy is tailored to the clinical variant of KS and disease stage. However, apart from highly active anti-retroviral therapy (HAART), treatment options are similar for the different epidemiologic forms of KS. The majority of patients with KS in sub-Saharan Africa present with advanced stage of the illness, and hence the aim of treatment is to control symptoms, palliate pain and improve their quality of life [23–25]). Surgical excision is restricted for cosmetically removing KS lesions, to alleviate discomfort. In patients with localised disease, localised treatment modalities are recommended and these include: cryotherapy, laser therapy intra-lesional chemotherapy, surgery and radiotherapy. All patients with AIDS-associated KS should initiate HAART as soon as the diagnosis is made, regardless of the CD4 T-cell counts. Treatment with HAART may induce complete remission in up to 80% of patients with good immunological response and limited disease [26]. The preferred first line chemotherapy in most low-resource countries is a combination of bleomycin and Vincristine, given every three weeks for six to nine cycles to achieve very good partial response or clinical remission. In contrast, liposomal doxorubicin is the preferred first-line regimen for AIDS-associated KS in high-income countries due to its higher efficacy and reduced toxicity [27].

Co-existing opportunistic infections, mainly TB are common among patients, with AIDS-related KS reflecting mainly the degree of immunosuppression, requiring appropriate treatment. Extensive lymphedema resulting from KSHV-induced exuberant proliferation of endothelial cells may lead to the occlusion of lymphatic vascular lumens leading to lymphedema and lymphadenopathy [28]. There is currently no clear strategy for management of lymphedema among KS patients.

## Conclusions

KS is a vascular tumour of endothelial origin that is associated with HHV8 infection. In sub-Saharan Africa, AIDS-KS remains the most common HIV-associated malignancy and hence it poses a huge burden to the already constrained health-care systems. KS has four clinical variants, namely, classic, endemic, iatrogenic and epidemic KS. The histopathology in these different KS forms is essentially identical; however, they have different clinical patterns. Even though there is no cure at present, the expanding knowledge of KS biology increases hope for prevention and control of the disease hence better quality of life among patients with KS. Primary prevention strategy for KSHV and management of disease complication such as lymphedema should be the focus of research.

## Conflicts of interest

The author has no conflicts of interest to declare.

## Funding declaration

This work was supported by the Uganda Cancer Institute.
